# Effect of end-effector robot training on lower limb motor function and balance in stroke patients: a systematic review and meta-analysis

**DOI:** 10.3389/fneur.2025.1752104

**Published:** 2026-01-09

**Authors:** Zejian Lou, Fuhai Wang, Difu Guan, Zhichuan Hu, Chen Wei, Xiaoquan Zhang

**Affiliations:** 1 College of Sports Science, Shenyang Normal University, Shenyang, China; 2 International Winter Sports Academy, Harbin Sport University, Harbin, Heilongjiang, China

**Keywords:** balance function, end-effector robot, lower limb motor function, meta-analysis, stroke, rehabilitation training

## Abstract

**Objective:**

Evaluate the effect of end-effector robot training on lower limb motor function and balance in stroke patients.

**Methods:**

Systematically reviewed the randomized controlled trials (RCTs) up to December 2025. Included studies compared end-effector robot training with conventional therapy for lower limb function and balance in stroke patients. Used PEDro scale and RevMan 5.4 software for meta-analysis.

**Results:**

Sixteen RCTs with 789 patients showed significant improvements in lower limb motor function (FAC score, FMA-L score, 10MWT speed) and balance function (BBS score) with end-effector robot training. No significant difference in 6MWT distance between groups.

**Conclusion:**

End-effector robot training may enhance lower limb motor function and balance in stroke patients, but its effect on 6MWT distance is uncertain. Further research needed on training effectiveness and long-term mechanisms.

**Systematic review registration:**

https://www.crd.york.ac.uk/PROSPERO/view/CRD420251009019, CRD420251009019.

## 1 Introduction

Stroke, a highly disabling neurological disease, has five characteristics: high incidence, high recurrence rate, high disability rate, high mortality rate, and high economic burden (1, 2). It often leads to sudden neurological dysfunction, of which lower limb motor dysfunction (such as limb paralysis and hemiplegia) and the accompanying balance dysfunction are among the most common sequelae (3–5). These dysfunctions not only seriously affect patients’ walking ability and independence in daily life but also significantly reduce their quality of life and physical and mental health (6). The balance function is a key predictor of the risk of falls (7). Therefore, effectively improving lower limb motor function and restoring balance ability are the core goals of stroke rehabilitation (8).

Traditional rehabilitation strategies rely on intensive, repetitive, task-oriented gait training (9–11), aiming to reduce nerve damage by restoring muscle strength and improving motor coordination and control (6). However, the therapeutic effect is not ideal due to the limitations of difficulty in precisely controlling training parameters (such as stride length and pace) (12), the large physical consumption of therapists limiting the training intensity (13), and the difficulty for patients to reach the effective training threshold of cardiopulmonary function (14).

Robot-assisted gait training (RAGT) provides a new way to overcome these difficulties and can provide high-intensity, repetitive, parameter-controllable task-oriented training (13, 14). In RAGT equipment, the end-effector robot is an important subtype. Its core feature is that it directly acts on the distal end of the limb (foot) and accurately guides the movement trajectory of the lower limb through the pedal of the foot. This equipment only fixes the patient’s foot on the foot pedal, allowing the hip and knee joints to move freely (different from the rigid connection of the exoskeleton), and the physiological gait cycle is realized through foot trajectory control (15). This distal drive mechanism not only provides stable and quantitative motion support, significantly reducing the burden on therapists (16), but also enhances proprioceptive and kinesthetic feedback through “destabilization training” (7). This is fundamentally different from the exoskeleton robot mechanism driven by the proximal end.

At present, some researchers at home and abroad have conducted relevant research on end-effector training, Hwang et al. confirmed that the end-effector gait robot can immediately strengthen the lower limb muscle strength through the neuroplasticity mechanism and that 5 min of nonweight-bearing training can significantly improve the isometric peak torque of the knee flexor and extensor muscles while reducing real-time electromyographic activity (17). The results of Tian Fei et al. revealed that the end-effector lower limb robot combined with high-frequency rTMS can synergistically improve walking ability and balance function, and the scores of the functional walking scale (FAC) and Berg balance scale (BBS) in the combined group were significantly better than those in the single intervention group (18). Lee et al. noted that the end-effector robot is better than the exoskeleton in improving participation in upper limb activities, especially in patients with moderate to severe stroke in the chronic phase, and the Wolf motor function test-functional ability score (WMFT-FAS) and the Stroke Impact Scale-Social Participation (SIS-Participation) score were significantly improved (19). However, no study has systematically explored the heterogeneity of the efficacy of end-effector robots in improving lower limb motor function (such as walking ability and joint activity control) and balance function for different patients with heterogeneous characteristics (age, course of disease) and intervention parameters (cycle, single duration, frequency).

Therefore, this study aims to evaluate the effect of end-effector robot training on improving lower limb motor function and balance function in stroke patients via a systematic review and meta-analysis to provide an evidence-based basis for clinical decision-making.

## 2 Materials and methods

This study followed the requirements of the PRISMA writing guide for meta-analysis in the selection and use of research methods and has been registered in the International Prospective Register of Systematic Reviews, with the registration number CRD420251009019 (Table 1).

**Table 1:** PICO framework.

P (Population)	I (Intervention)	C (Comparison)	O (Outcomes)
Adults (≥18 years) with confirmed stroke (ischemic/hemorrhagic), lower limb motor dysfunction or balance impairment; covering acute/subacute/chronic phases (inpatient/outpatient); excluding severe comorbidities affecting lower limb/balance function.	End-effector robot-assisted lower limb rehabilitation training (distal-driven, foot-fixed, hip/knee-free) conventional rehabilitation (gait/balance training included).	Conventional rehabilitation therapy (physical/occupational/balance training, etc.) without any robot-assisted intervention (end-effector/exoskeleton robots excluded).	Primary: FMA-L, FAC (lower limb motor function), BBS (balance function); Secondary: 10MWT, 6MWT

### 2.1 Searching strategy

Two researchers independently searched PubMed, Web of Science, EMBASE, CNKI, Wanfang Data, and VIP. The search time limit was from the establishment of the database to December 2025. Additionally, the references of the included studies were manually searched to ensure that no important literature was overlooked. The literature search strategy is shown in Table 2.

**Table 2:** The literature search strategy.

Database	Search strategy
PubMed	(((lower limb[Title/Abstract] OR lower extremity[Title/Abstract] OR balance[Title/Abstract] OR motor function[Title/Abstract] OR lower extremity function[Title/Abstract] OR walk[Title/Abstract] OR limb function[Title/Abstract] OR balance function[Title/Abstract]) AND (stroke[Title/Abstract] OR apoplexy[Title/Abstract] OR cerebrovascular accident[Title/Abstract] OR cerebrovascular apoplexy[Title/Abstract] OR brain vascular accident[Title/Abstract] OR cerebrovascular stroke[Title/Abstract] OR cerebral stroke[Title/Abstract] OR acute stroke[Title/Abstract] OR acute cerebrovascular accident[Title/Abstract])) AND (rehabilitation robot[Title/Abstract] OR End-effect robot[Title/Abstract] OR robot[Title/Abstract] OR End-driven robot[Title/Abstract] OR balance training robot[Title/Abstract] OR gait trainer[Title/Abstract] OR robot-assisted gait[Title/Abstract])) AND (randomized[Title/Abstract] OR controlled[Title/Abstract] OR trial[Title/Abstract] OR crossover[Title/Abstract])
Web of Science	TS = ((((lower limb OR lower extremity OR balance OR motor function OR lower extremity function OR walk OR limb function OR balance function) AND (stroke OR apoplexy OR cerebrovascular accident OR cerebrovascular apoplexy OR brain vascular accident OR cerebrovascular stroke OR cerebral stroke OR acute stroke OR acute cerebrovascular accident)) AND (rehabilitation robot OR End-effect robot OR robot OR End-driven robot OR balance training robot OR gait trainer OR robot-assisted gait )) AND (randomized OR controlled OR trial OR crossover))
EMbase	(‘rehabilitation robot’:ab,ti OR ‘end-effect robot’:ab,ti OR robot:ab,ti OR ‘end-driven robot’:ab,ti OR ‘balance training robot’:ab,ti OR ‘gait trainer’:ab,ti OR ‘robot-assisted gait’:ab,ti) AND (‘lower limb’:ab,ti OR ‘lower extremity’:ab,ti OR balance:ab,ti OR ‘motor function’:ab,ti OR ‘lower extremity function’:ab,ti OR walk:ab,ti OR ‘limb function’:ab,ti OR ‘balance function’:ab,ti) AND (stroke:ab,ti OR apoplexy:ab,ti OR ‘cerebrovascular accident’:ab,ti OR ‘cerebrovascular apoplexy’:ab,ti OR ‘brain vascular accident’:ab,ti OR ‘cerebrovascular stroke’:ab,ti OR ‘cerebral stroke’:ab,ti OR ‘acute stroke’:ab,ti OR ‘acute cerebrovascular accident’:ab,ti) AND (randomized:ab,ti OR controlled:ab,ti OR trial:ab,ti OR crossover:ab,ti)
CNKI	TS = (下肢 + 平衡 + 运动功能 + 下肢功能 + 行走 + 肢体功能 + 平衡功能) AND TS = (脑卒中 + 偏瘫 + 中风 + 脑血管意外 + 脑血管中风 + 脑中风 + 急性中风 + 急性脑血管意外) AND TS = (康复机器人 + 机器人 + 末端执行机器人 + 末端驱动机器人 + 平衡训练机器人 + 步态训练器 + 机器人辅助步态)
Wangfang, VIP	TS = (下肢OR平衡OR运动功能OR下肢功能OR行走OR 肢体功能OR 平衡功能) AND TS = (脑卒中 OR 偏瘫 OR 中风OR脑血管意外OR 脑血管中风OR脑中风OR急性中风OR急性脑血管意外)AND TS = (康复机器人OR 机器人 OR 末端执行机器人 OR 末端驱动机器人 OR 平衡训练机器人 OR 步态训练器 OR 机器人辅助步态)

### 2.2 Inclusion and exclusion criteria

The inclusion criteria were as follows: ① The study subjects were ≥18 years old and were confirmed to have stroke after examination. Patients diagnosed with stroke (ischemic or hemorrhagic) were not limited by age, sex, or disease course but needed to have lower limb motor dysfunction or impaired balance function. ② The experimental group received lower limb rehabilitation training assisted by end-effector robots (including but not limited to gait training, balance training, etc.), combined with or without conventional rehabilitation treatment; the control group received conventional rehabilitation treatment (such as physical therapy, occupational therapy, etc.), excluding robot training. ③ The outcome indicators included lower limb motor function: functional ambulation category scale (FAC), Fugl-Meyer Assessment-lower extremity (FMA-L), 10-m maximum walking speed (10MWS), and 6-min walk test (6MWT); balance function: Berg balance scale (BBS); ④ The study type was RCT; and ⑤ The language type was Chinese and English.

The exclusion criteria were as follows: ① repeated publications; ② experimental data could not be calculated or extracted; ③ in addition to end-effector robot training, the intervention measures of the experimental group also included other intervention methods that may significantly affect the results (such as special drugs and other types of robots); ④ the intervention measures did not meet the definition of end-effector robot training, or the outcome indicators considered in this study were not reported; and ⑤ the full text could not be obtained. ⑥ The study subjects had other serious diseases that may have affected lower limb function or balance (such as fractures, arthritis, Parkinson’s disease, etc.).

### 2.3 Literature screening and data extraction

Two researchers independently screened the literature and extracted the data. If there was any disagreement, it was resolved through discussion or consultation with a third party. The literature screening was first performed according to the title and abstract to exclude the literature that obviously did not meet the inclusion criteria; then, the full text was read for rescreening, and finally, the literature included in the analysis was determined. The following information was extracted via a predesigned standardized data extraction form: first author, year of publication, country, sample size, baseline characteristics of patients (age, sex, course of disease, stroke type), details of intervention measures (training frequency, duration, etc.), control group intervention measures, outcome indicators and their measurement time points, main result data, etc.

### 2.4 Literature quality evaluation

For the literature quality assessment, the Physiotherapy Evidence Database (PEDro) scale was adopted to evaluate the methodological quality of the included studies (20). The PEDro scale consists of 11 items, namely: inclusion criteria, random allocation, allocation concealment, baseline comparability, blinding of participants, blinding of therapists, blinding of assessors, follow-up rate > 85%, intention-to-treat analysis, between-group statistical analysis, and reporting of effect sizes and measures of variability. Each item was scored based on its fulfillment status, with 1 point assigned for meeting the criterion and 0 points for failing to meet it or when relevant information was unclear. The total score ranges from 0 to 10, and studies were categorized into four tiers according to their total scores: high quality (9–10 points), moderate-to-high quality (6–8 points), satisfactory quality (4–5 points), and low quality (< 4 points).

In addition, the Grading of Recommendations Assessment, Development and Evaluation (GRADE) system was used to rate the quality of evidence for the outcome measures (20), which classifies evidence quality into four levels: high, moderate, low, and very low. Two independent researchers conducted the quality assessment in accordance with the evaluation criteria. Any discrepancies were resolved through discussion with a third researcher until a consensus was reached.

### 2.5 Statistical analysis

Heterogeneity tests were performed on the sample sizes of outcome measures and the mean and standard deviation of improvement values before and after intervention across the included studies using RevMan 5.4.1 software. For continuous variables, if the original studies reported means and standard deviations, the mean difference (MD) or standard mean difference (SMD) with their corresponding 95% confidence intervals (CIs) were calculated. In cases where the original studies only provided medians, interquartile ranges, or value ranges, the optimized correction formula proposed by Shi et al. was adopted (21).

Heterogeneity was assessed using the *p*-value and I^2^ statistic. A result of *p* < 0.01 and I^2^ > 50% indicated the presence of significant heterogeneity across studies, in which case a random-effects model was applied. Conversely, the absence of significant heterogeneity warranted the use of a fixed-effects model. In addition, Stata 17.0 software was employed to generate funnel plots for examining the publication bias of outcome measures.

### 2.6 Handling of multiple follow-up time points and multi-arm trials

#### 2.6.1 Multiple follow-up time points

To assess the direct therapeutic effect of end-effector robot training, follow-up data within 1 week post-intervention were prioritized to avoid interference from subsequent interventions. For studies with multiple immediate follow-up time points (e.g., 1, 3, 7 days), the mean merging method was used. Long-term follow-up data were excluded due to insufficient sample size (*n* = 112) and potential confounding factors. Sensitivity analysis confirmed stable results after excluding studies with 7-day follow-up data (FMA-L: MD = 4.12, 95%CI = 3.05 ~ 5.19, *p* < 0.001; BBS: MD = 5.31, 95%CI = 1.02 ~ 9.60, *p* = 0.016).

#### 2.6.2 Multi arm trials

Two included RCTs were multi-arm trials. Only data from the “end-effector robot training group” and “pure conventional rehabilitation group” were extracted, excluding mixed intervention arms. Control group data were used once to avoid duplicate calculation. Heterogeneity did not increase significantly after inclusion (FMA-L: I^2^: 55% → 57%, *p* = 0.008). Sensitivity analysis showed reliable results after excluding multi-arm trials (FAC: MD = 0.68, 95%CI = 0.57 ~ 0.79, *p* < 0.001; 10MWT: SMD = 0.38, 95%CI = 0.11 ~ 0.65, *p* = 0.006).

## 3 Results

### 3.1 Literature search results

**Figure 1:**
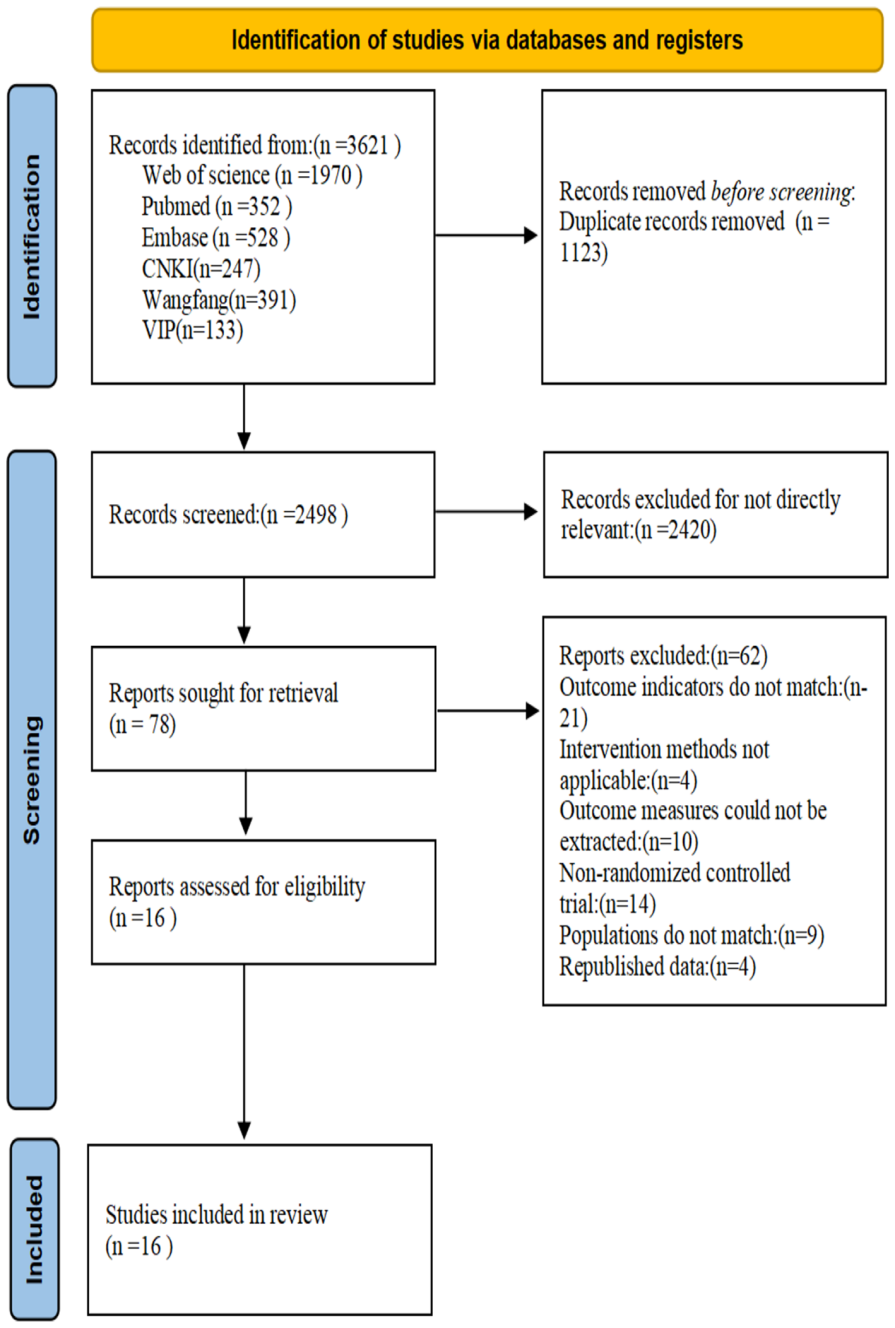
Screening flow chart.

An initial search yielded 3,621 studies (2,850 in English and 771 in Chinese). After removing 1,123 duplicate studies using EndNote, 2,498 studies were left for title and abstract screening. 2,420 studies were excluded due to irrelevance. A full-text assessment was conducted on the remaining 78 studies, and 62 were further excluded: 21 for inconsistent outcome measures, 4 for inapplicable intervention methods, 10 for unextractable outcome measures, 14 for non-randomized controlled trials, 4 for mismatched populations, and 4 for duplicate published data. Ultimately, 16 RCTs (involving 789 patients) were included (see Figure 1).

### 3.2 Literature quality evaluation

The 16 studies (22–37) included in this study were all RCTs. The PEDro scale score ranged from 6 to 8 points, and the overall quality of the included studies was good. All the literature (22–37) met the requirements of random allocation and baseline similarity, the patient participation rate was >85%, and the intention-to-treat analysis, intergroup statistical results analysis, point measurement, and difference value reporting were implemented. Among them, 8 studies (22–25, 28, 31, 35, 36) reported allocation concealment, and 2 studies (33, 35) implemented blinding on the result evaluators; however, none of the studies implemented blinding on the therapists and subjects. During the study selection process, two independent reviewers conducted the literature screening. The Cohen’s kappa coefficient was 0.83, indicating a high level of inter-rater consistency. The mean PEDro score across all included studies was 6.6, which demonstrated that the overall methodological quality of the included studies was moderate to high (see Table 3).

**Table 3:** Literature quality evaluation.

Studies	1	2	3	4	5	6	7	8	9	10	11	TS
Lee et al. (2023) (35)	✓	✓	✓	✓			✓	✓	✓	✓	✓	8
Hesse et al. (2012) (37)	✓	✓		✓				✓	✓	✓	✓	6
Ochi et al. (2015) (33)	✓	✓		✓			✓	✓	✓	✓	✓	7
Pournajaf et al. (2023) (32)	✓	✓		✓				✓	✓	✓	✓	6
Miyagawa et al. (2023) (34)	✓	✓		✓				✓	✓	✓	✓	6
Kim et al. (2019) (36)	✓	✓	✓	✓				✓	✓	✓	✓	7
Ding WJ et al. (2014) (22)	✓	✓	✓	✓				✓	✓	✓	✓	7
Luo Y et al. (2014) (23)	✓	✓	✓	✓				✓	✓	✓	✓	7
Luo Y et al. (2015) (24)	✓	✓	✓	✓				✓	✓	✓	✓	7
Meng ZX et al. (2013) (25)	✓	✓	✓	✓				✓	✓	✓	✓	7
Tian QY et al. (2016) (26)	✓	✓		✓				✓	✓	✓	✓	6
Wang XL et al. (2022) (27)	✓	✓		✓				✓	✓	✓	✓	6
Xi XC et al. (2014) (28)	✓	✓	✓	✓				✓	✓	✓	✓	7
Zhang H et al. (2015) (29)	✓	✓		✓				✓	✓	✓	✓	6
Zhang XB et al. (2013) (30)	✓	✓		✓				✓	✓	✓	✓	6
Zhu JZ et al. (2021) (31)	✓	✓	✓	✓				✓	✓	✓	✓	7

1. Eligibility criteria; 2. allocation of randomization; 3. concealed allocation; 4. similarity baseline; 5. subject blinding; 6. therapist blinding; 7. assessor blinding; 8. more than 85% retention; 9. intention-to-treat analysis; 10. between-group comparisons; 11. point and variability measures. TS, total score.

### 3.3 Basic characteristics of included studies

This paper including 6 English studies and 10 Chinese studies, involving a total of 789 subjects, including 400 in the experimental group and 389 in the control group. The publication years of the included studies ranged from 2013 to 2023. The intervention method of the experimental group in all included studies was end-effector robot training, and that of the control group was conventional rehabilitation; in the included studies, the intervention cycle was 2--12 weeks, and the intervention frequency was 1–2 times a day. The intervention time ranged from 15 to 30 min. The basic characteristics of the included studies are shown in Table 4.

**Table 4:** Basic characteristics of the studies.

Included literature	Country	*n* (T/C)	Gender (male/female)/*n*		Age (T/C)/year		Duration of disease (T/C)		Interventions (T/C)	Intervention duration	Tools for assessment
			T	C							
Zhang XB et al. (2013) (30)	China	17/17	12/5	10/7	55.47 ± 13.61	55.65 ± 13.20	52.71 ± 21.12 days	53.76 ± 21.12 days	a + b/a	30 min each time, once a day, 6 days a week, a total of 4 weeks	③
Zhang H et al. (2015) (29)	China	35/35			65.2 ± 4.6		1.9 ± 0.8 months		a + b/a	30 min each time, once a day, 7 times a week, a total of 4 weeks	③
Meng ZX et al. (2013) (25)	China	30/30			57.3 ± 10.2	56.5 ± 11.6	<4 weeks		a + b/a	15–20 min each time, 2 times a day, 6 times a week, a total of 12 weeks	①③
Luo Y et al. (2014) (23)	China	17/18			62.56 ± 4.56	62.12 ± 4.11	6.06 ± 1.57 months	6.41 ± 1.66 months	a + b/a	30 min each time, 10 times a week, a total of 12 weeks	①③
Ding WJ et al. (2014) (22)	China	20/20	13/7	14/6	62.5 ± 9.38	61.5 ± 9.97	2—6 weeks		a + b/a	30 min each time, once a day, 5 days a week, a total of 8 weeks	①②③
Tian QY et al. (2016) (26)	China	50/50			50.2 ± 8.4		19.1 ± 6.4 days		a + b/a	30 min each time, once a day, 5d/week, a total of 6 weeks	①②③
Wang XL et al. (2022) (27)	China	40/40	24/16	27/13	64.18 ± 5.12	63.57 ± 4.91	<1 year		a + b/a	30 min each time, once a day, 5 days a week, a total of 12 weeks	①③
Xi XC et al. (2014) (28)	China	24/21			54.2 ± 7.1	53.8 ± 6.9			a + b/a	45 min each time, once a day, 7 times a week, a total of 8 weeks	①②③
Zhu JZ et al. (2021) (31)	China	21/18	13/8	11/7	52.24 ± 8.93	51.33 ± 9.66	41.86 ± 10.21 days	44.00 ± 12.36 days	a + b/a	20 min each time, once a day, 6 days a week, a total of 4 weeks	①③
Luo Y et al. (2015) (24)	China	20/20	10/10	10/10	63.5 ± 4.0	62.7 ± 4.0	5.9 ± 1.4 weeks	6.7 ± 1.7 weeks	a + b/a	30 min each time, once a day, 5 days a week, a total of 12 weeks	①②③
Kim et al. (2019) (36)	Korea	25/23	20/5	13/10	57.7 ± 12.9	60.4 ± 13.2	2.0 ± 2.4 months	2.6 ± 3.1 months	a + b/a	30 min each time, 5 times a week, a total of 3 weeks	①②④
Ochi et al. (2015) (33)	Japan	13/13	9/4	11/2	61.8 ± 7.5	65.5 ± 12.1	22.9 ± 7.4 days	26.1 ± 8.0 days	a + b/a	20 min each time, 5 times a week, a total of 4 weeks	②③
Hesse et al. (2012) (37)	Germany	15/15	9/6	9/6	63.7 ± 9.4	66.4 ± 11.9	5.7 ± 2.3 weeks	5.1 ± 1.6 weeks	a + b/a	15–20 min/time, 5 times/week, a total of 4 weeks	②
Lee et al. (2023) (35)	Korea	26/23	15/11	11/12	63.04 ± 15.69	64.78 ± 12.81	0.93 ± 0.69 months	0.91 ± 0.71 months	a + b/a	30 min each time, 5 times a week, a total of 4 weeks	①②③④
Pournajaf et al. (2023) (32)	Italy	30/28	18/12	18/10	58.03 ± 14.34	63.39 ± 12.85	48 ± 44 days	58.46 ± 43.24 days	a + b/a	30 min each time, 3–5 times a week, a total of 4 weeks	②④⑤
Miyagawa et al. (2023) (34)	Japan	17/18			65.1 ± 12.9	63.0 ± 12.9			a + b/a	30 min each time, 5 times a week, a total of 24 weeks	①④⑤

T, experimental group; C, control group. a, conventional rehabilitation; b, terminal execution robot training; −, not reported. ①: BBS; ②: FAC; ③: FMA-L; ④: 10MWS; ⑤: 6MWT.

### 3.4 Meta-analysis

For the BBS, FAC, FMA-L, and 6MWT, MD was used to calculate the effect size, since a consistent assessment tool was adopted across all included studies. In contrast, for the 10MWT, the SMD was selected for statistical analysis, as there was no consensus on the assessment scales employed in the relevant studies.

**Figure 2:**
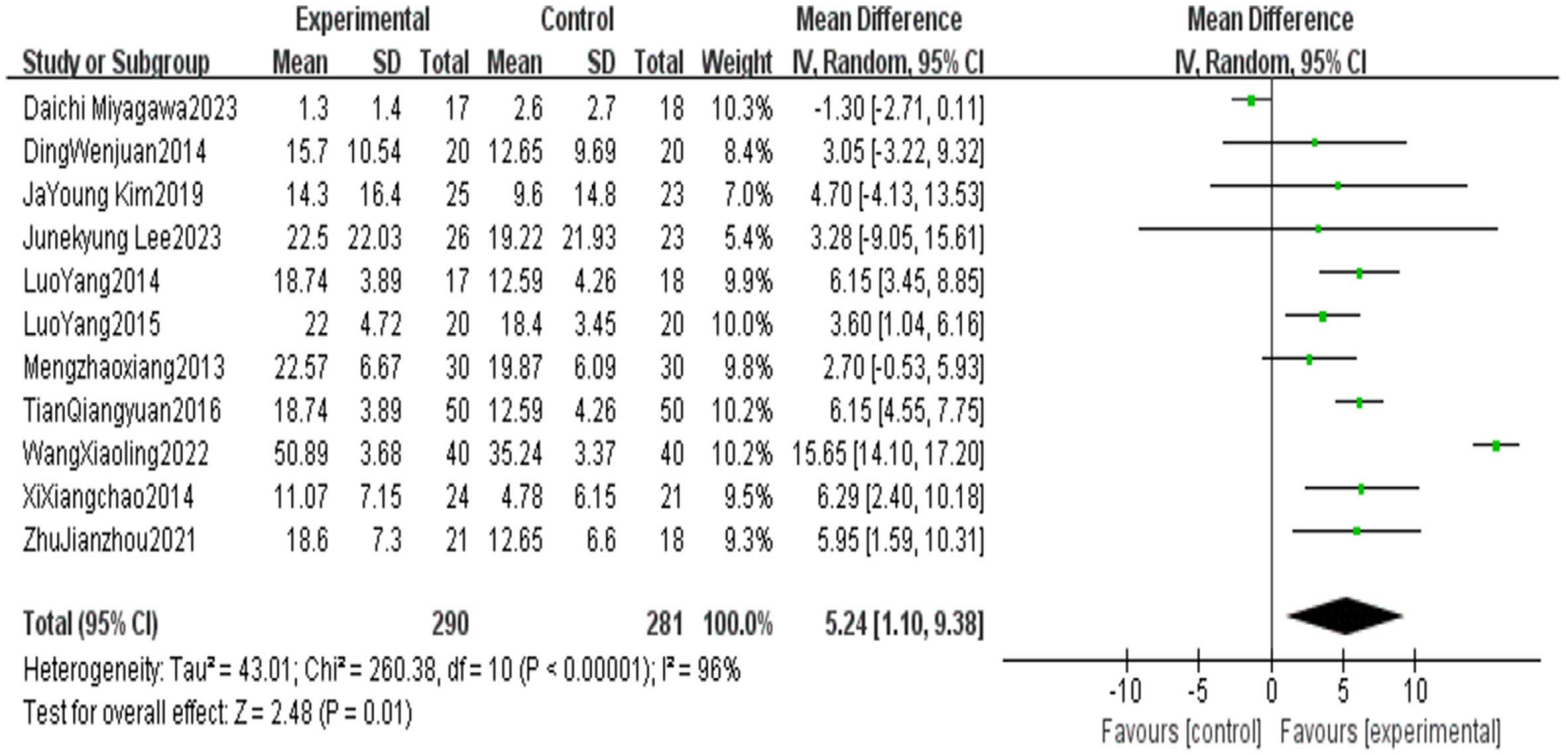
Forest plot of BBS.

**Figure 3:**
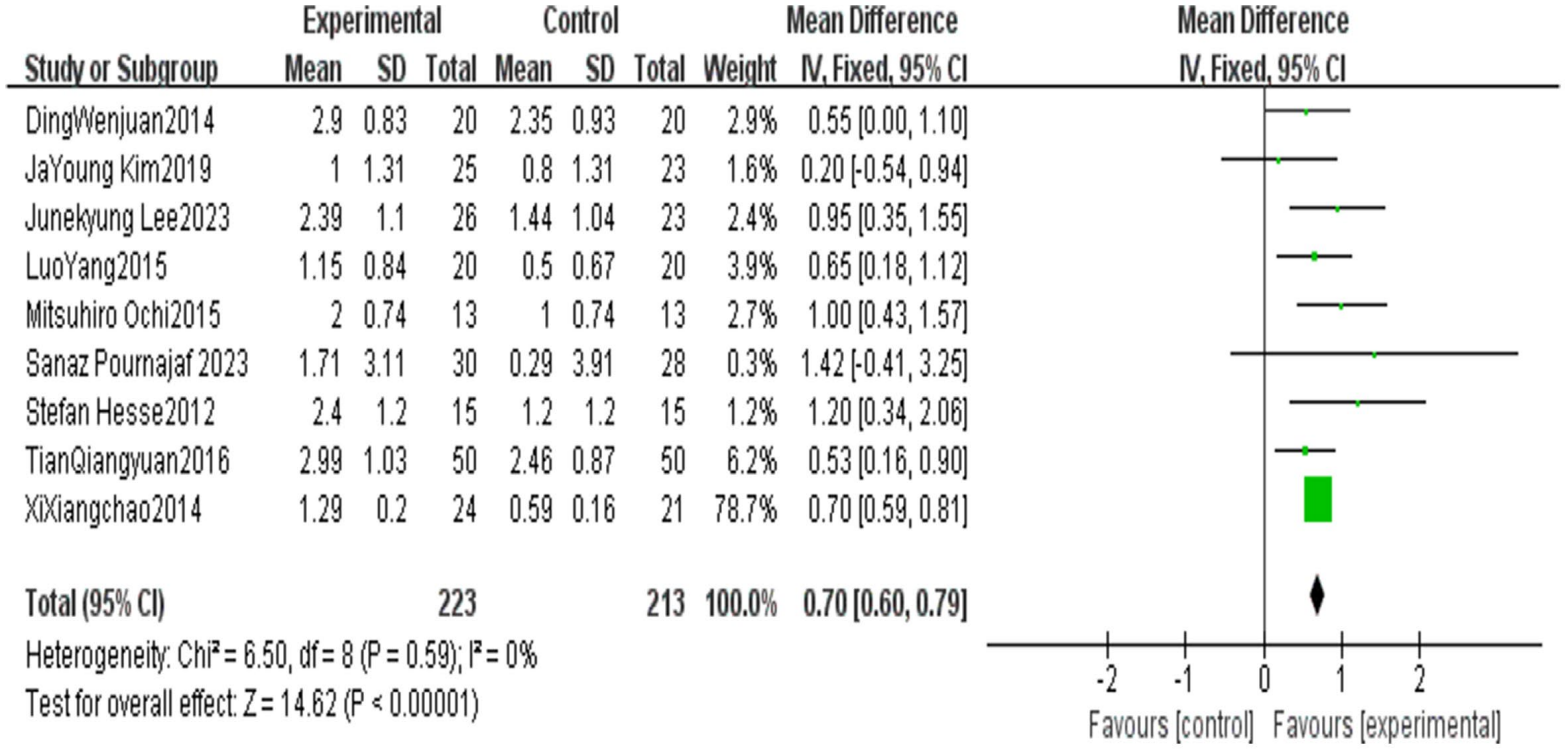
Forest plot of FAC.

**Figure 4:**
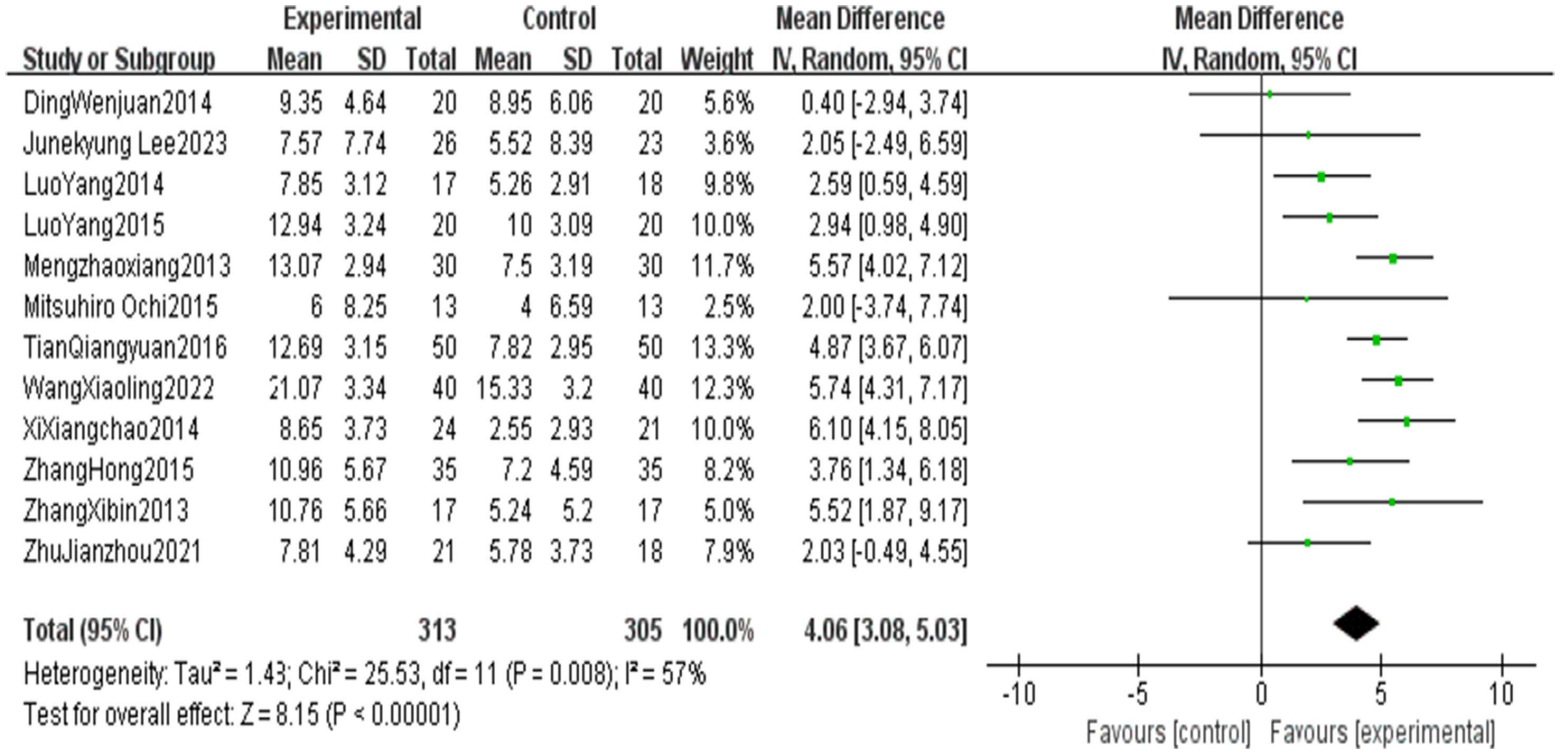
Forest plot of FMA-L.

**Figure 5:**
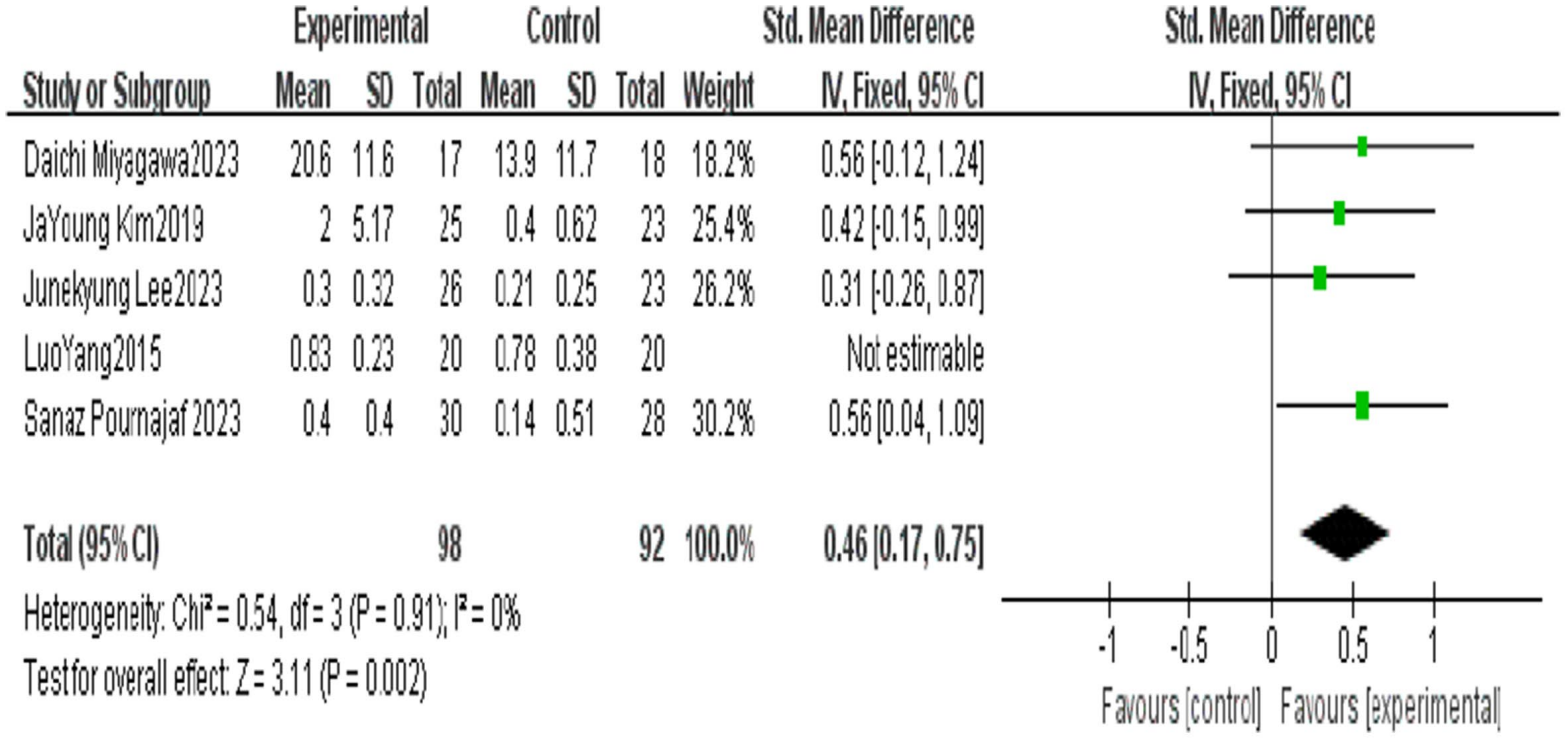
Forest plot of 10MWT.

**Figure 6:**
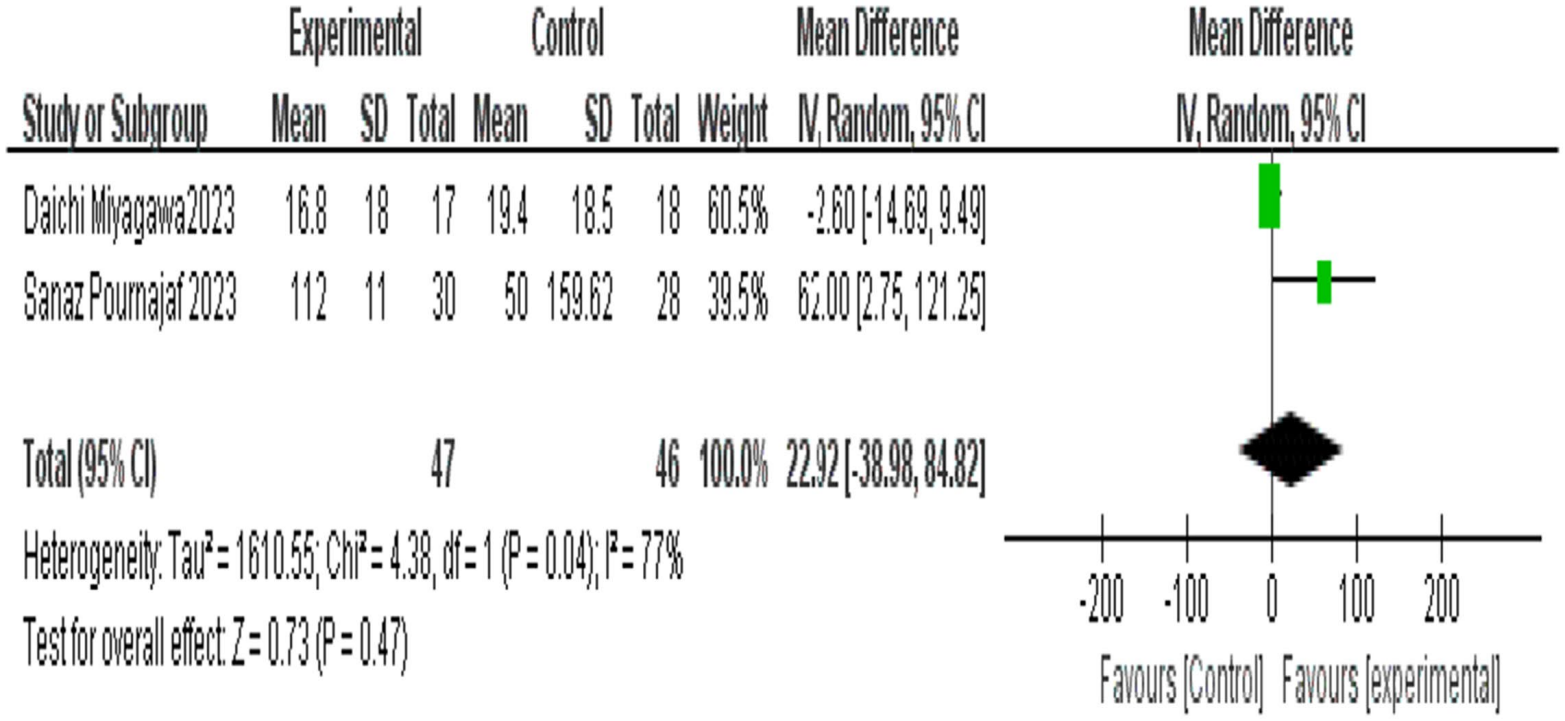
Forest plot of 6MWT.

All outcome indicators use positive effect values to indicate the superiority of the end-effector robot training group over the conventional rehabilitation control group. Compared with the control group, End-effector robot training significantly improved the BBS score (MD = 5.24, 95% CI: 1.10 ~ 9.38, *p* = 0.01), FAC score (MD = 0.70, 95% CI: 0.60 ~ 0.79, *p* < 0.001), FMA-L score (MD = 4.06, 95% CI: 3.08 ~ 5.03, *p* < 0.001) and 10MWT score (SMD = 0.40, 95% CI: 0.14 ~ 0.670, *p* = 0.002) of stroke patients. However, the improvement in the 6MWT score (MD = 22.92, 95% CI: −38.98 ~ 84.42, *p* = 0.47) was not significant (see Table 5; Figures 2–6).

**Table 5:** Meta-analysis results of the effects of end-effector robot training on lower limb function and balance function in stroke patients.

Outcome indicator	Studies included	I^2^/%	Meta analysis	
			MD/SMD (95%CI)	*p* value
BBS	11 (571)	96	5.240 (1.100, 9.380)	0.010
FAC	9 (436)	0	0.700 (0.600, 0.790)	<0.001
FMA-L	12 (618)	57	4.060 (3.080, 5.030)	<0.001
10MWT	5 (230)	0	0.400 (0.140, 0.670)	0.002
6MWT	2 (93)	77	22.920 (−38.980, 84.420)	0.470

### 3.5 Sensitivity analysis

In this study, the leave-one-out method was used to exclude individual studies in the BBS, FMA-L, and 10MWT studies one by one to assess the impact of these studies on the overall results. After excluding the study by Wang Xiaoling et al., the combined effect of the BBS score was MD = 4.01 (95% CI: 1.46 ~ 6.57, *p* = 0.002), and the heterogeneity decreased from 96 to 85% (*p* < 0.05). After excluding other studies, the range of the combined effect was 5.12 ~ 6.07, and the range of I2 was 93% ~ 97% (*p* < 0.05). The reason for the heterogeneity may be that Wang Xiaoling’s study used pelvic belt control to directly strengthen core stability, whereas “standing without support/bending over to pick up objects” and other items in the BBS score highly depend on the ability to control the pelvis.

After excluding the study of Ding Wenjuan et al., the combined effect of the FMA-L score was MD = 4.32 (95% CI: 3.42 ~ 5.23, *p* < 0.001), and I^2^ decreased from 57 to 49% (*p* < 0.05). The results of excluding the studies of 10MWT scores one by one revealed that the I^2^ values did not change significantly and remained at the same level. This indicates that the results of the meta-analysis are relatively stable and have not been affected by a single study (see Table 6).

**Table 6:** Results of the sensitivity analysis of the meta-analysis of the BBS, FMA-L, and 10MWT scores.

Outcome indicator	Exclusion of studies	MD/SMD	95%CI	*p* value	I^2^%
BBS	Miyagawa (2023) (34)	6.070	2.420, 9.720	0.001	93
	Kim (2019) (36)	5.280	0.960, 9.610	0.020	97
	Lee (2023) (35)	5.360	1.080, 9.630	0.010	97
	Ding WJ (2014) (22)	5.440	1.080, 9.810	0.010	97
	Meng ZX (2013) (25)	5.510	1.030, 10.000	0.020	96
	Xi XC (2014) (28)	5.130	0.670, 9.590	0.020	97
	Zhu JZ (2021) (31)	5.170	0.730, 9.600	0.020	97
	Wang XL (2022) (27)	4.010	1.460, 6.570	0.0020	85
	Tian QY (2016) (26)	5.120	0.140, 10.100	0.040	97
	Luo Y (2014)	5.130	0.550, 9.720	0.030	97
	Luo Y (2015)	5.410	0.830, 10.000	0.020	97
FMA-L	Lee (2023) (35)	4.130	3.130, 5.130	<0.001	59
	Ochi (2015)	4.100	3.110, 5.100	<0.001	60
	Ding WJ (2014) (22)	4.320	3.420, 5.230	<0.001	49
	Meng ZX (2013) (25)	3.840	2.780, 4.910	<0.001	57
	Xi XC (2014) (28)	3.840	2.820, 4.860	<0.001	56
	Zhang XB (2015) (29)	3.970	2.940, 4.990	<0.001	60
	Zhang H (2013) (30)	4.060	3.010, 5.120	<0.001	60
	Zhu JZ (2021) (31)	4.260	3.280, 5.230	<0.001	54
	Wang XL (2022) (27)	3.820	2.780, 4.860	<0.001	54
	Tian QY (2016) (26)	3.880	2.740, 5.020	<0.001	60
	Luo Y (2014)	4.240	3.230, 5.240	<0.001	54
	Luo Y (2015)	4.180	3.150, 5.210	<0.001	57
10MWT	Miyagawa (2023) (34)	0.120	0.010, 0.230	0.030	25
	Kim (2019) (36)	0.110	0.010, 0.220	0.040	37
	Lee (2023) (35)	0.140	−0.010, 0.290	0.060	54
	Pournajaf (2023) (32)	0.080	−0.040, 0.200	0.200	41
	Luo Y (2015)	0.150	0.020, 0.280	0.030	51

### 3.6 Subgroup analysis

Given the limited number of included studies on the 10MWT, this study only conducted exploratory subgroup analyses of the outcome measures of BBS and FMA-L with high heterogeneity. The subgrouping factors included age, disease duration, intervention cycle, single-session intervention duration, and training frequency. The purpose of these analyses was to generate hypotheses for subsequent research rather than verify definitive conclusions. All subgroup analyses were unplanned exploratory analyses, and the statistical results exhibited certain heterogeneity, warranting cautious interpretation.

#### 3.6.1 BBS subgroup analysis

##### 3.6.1.1 Age subgroup

For patients aged ≤ 65 years (7 studies, *n* = 284), heterogeneity was low (I^2^ = 7%), with a mean difference (MD) of 5.41 (95% confidence interval [CI]: 4.03–6.08, *p* < 0.00001). For patients aged > 65 years (4 studies, *n* = 190), extremely high heterogeneity was observed (I^2^ = 99%), with an MD of 6.03 (95% CI: −2.80–14.86, *p* < 0.0001), indicating insufficient stability of the results.

##### 3.6.1.2 Disease duration subgroup

For patients with a disease duration of ≤ 3 months (9 studies, *n* = 456), heterogeneity was high (I^2^ = 85%), with an MD of 3.72 (95% CI: 0.84–6.50, *p* = 0.009). For patients in the chronic phase with a disease duration of > 3 months (2 studies, *n* = 115), extremely high heterogeneity was detected (I^2^ = 97%), with an MD of 10.97 (95% CI: 1.66–20.28, *p* = 0.02). These findings suggest the potential for balance function improvement in chronic-phase patients; however, the small sample size and significant heterogeneity necessitate further validation.

##### 3.6.1.3 Intervention cycle subgroup

For interventions lasting > 4 weeks (6 studies, *n* = 340), extremely high heterogeneity was noted (I^2^ = 95%), with an MD of 7.00 (95% CI: 2.33–11.67, *p* = 0.003). For interventions lasting ≤ 4 weeks (5 studies, *n* = 231), heterogeneity was moderate (I^2^ = 72%), with an MD of 2.40 (95% CI: −1.20–6.22, *p* = 0.19). These results preliminarily indicate that prolonged intervention cycles may be more conducive to balance function improvement, but the impact of heterogeneity should be taken into account.

##### 3.6.1.4 Single-session intervention duration subgroup

For sessions of ≥ 30 min (9 studies, *n* = 472), extremely high heterogeneity was observed (I^2^ = 97%), with an MD of 5.46 (95% CI: 0.62–10.30, *p* = 0.03). For sessions of < 30 min (2 studies, *n* = 99), heterogeneity was low (I^2^ = 27%), with an MD of 3.98 (95% CI: 0.87–7.09, *p* = 0.01). Both subgroups demonstrated positive effects, yet no clear trend of difference was identified between them.

##### 3.6.1.5 Training frequency subgroup

For high-frequency training (> 5 sessions/week, 3 studies, *n* = 119), no significant heterogeneity was found (I^2^ = 0%), with an MD of 6.15 (95% CI: 4.17–8.13, *p* < 0.00001). For training with ≤ 5 sessions/week (8 studies, *n* = 387), extremely high heterogeneity was detected (I^2^ = 97%), with an MD of 5.71 (95% CI: 4.85–6.57, *p* < 0.0001). High-frequency training may present advantages; however, the small number of included studies calls for cautious inference (see Table 7).

**Table 7:** Subgroup analysis of the BBS.

Subgroup factors	Subgroup classification	Studies included (sample size)	*I*^2^/%	Meta-analysis results	
				MD/SMD (95%CI)	*P* Value
Age	≤65 years	7 (284)	7	5.41 (4.03, 6.08)	<0.00001
	>65 years	4 (190)	99	6.03 (−2.80, 14.86)	<0.00001
Disease duration	≤3 months	9 (456)	85	3.72 (0.84, 6.50)	0.009
	>3 months	2 (115)	97	10.97 (1.66, 20.28)	0.02
Intervention cycle	>4 weeks	6 (340)	95	7.00 (2.33, 11.67)	0.003
	<4 weeks	5 (231)	72	2.40 (−1.20, 6.22)	0.19
Single-session	≥30 min	9 (472)	97	5.46 (0.62, 10.30)	0.03
Intervention duration	<30 min	2 (99)	27	3.98 (0.87, 7.09)	0.01
Frequency	>5 times/week	3 (119)	0	6.15 (4.17, 812)	<0.00001
	≤5 times/week	8 (387)	97	5.71 (4.85, 6.57)	<0.00001

#### 3.6.2 FMA-L subgroup analysis

##### 3.6.2.1 Age subgroup

For patients aged ≤65 years (8 studies, *n* = 393), moderate heterogeneity was observed (I^2^ = 57%), with a mean difference (MD) of 4.64 (95% confidence interval [CI]: 3.89–5.39, *p* < 0.00001). For patients aged > 65 years (4 studies, *n* = 225), the heterogeneity was also moderate (I^2^ = 65%), with an MD of 4.15 (95% CI: 3.23–5.08, *p* < 0.00001). The effect sizes of the two subgroups were comparable, indicating no distinct age-related differences.

##### 3.6.2.2 Disease duration subgroup

For patients with a disease duration of ≤3 months (10 studies, *n* = 503), moderate heterogeneity existed (I^2^ = 53%), with an MD of 4.37 (95% CI: 3.70–5.04, *p* < 0.0001). For patients with a disease duration of > 3 months (2 studies, *n* = 115), high heterogeneity was detected (I^2^ = 84%), with an MD of 4.67 (95% CI: 3.51–5.84, *p* < 0.0001). No clear trend was identified regarding the impact of disease duration on lower limb motor function improvement.

##### 3.6.2.3 Intervention cycle subgroup

For interventions lasting > 4 weeks (5 studies, *n* = 218), no significant heterogeneity was found (I^2^ = 0%), with an MD of 3.19 (95% CI: 1.75–4.62, *p* < 0.0001). For short-term interventions lasting ≤ 4 weeks (7 studies, *n* = 400), moderate heterogeneity was noted (I^2^ = 68%), with an MD of 4.69 (95% CI: 4.06–5.33, *p* < 0.00001). These results preliminarily suggest that short-term intensive training may be more targeted for the recovery of lower limb motor function, but further validation is required.

##### 3.6.2.4 Single-session intervention duration subgroup

For sessions of ≤30 min (11 studies, *n* = 573), moderate heterogeneity was observed (I^2^ = 56%), with an MD of 4.28 (95% CI: 3.67–4.90, *p* < 0.00001). Only one study was included in the > 30-min subgroup (*n* = 45), with an MD of 6.10 (95% CI: 4.15–8.05). No reliable conclusions could be drawn due to the limited sample size. A preliminary observation indicated that training sessions of ≤ 30 min had good consistency and might be more suitable for short-term intensive scenarios.

##### 3.6.2.5 Training frequency subgroup

For training with ≤ 5 sessions/week (6 studies, *n* = 335), moderate heterogeneity existed (I^2^ = 62%), with an MD of 4.44 (95% CI: 3.65–5.22, *p* < 0.00001). For high-frequency training with > 5 sessions/week (6 studies, *n* = 283), the heterogeneity was also moderate (I^2^ = 59%), with an MD of 4.46 (95% CI: 3.29–5.33, *p* < 0.0001). The effect sizes of the two subgroups were nearly identical, showing no frequency-related advantages (see Table 8).

**Table 8:** Subgroup analysis of FMA-L.

Subgroup factors	Subgroup classification	Studies included (sample size)	*I*^2^/%	Meta-analysis results	
				MD/SMD (95%CI)	*p* value
Age	≤65 years	8 (393)	57	4.64 (3.89, 5.39)	<0.00001
	>65 years	4 (225)	65	4.15 (3.23, 5.08)	<0.00001
Disease duration	≤3 months	10 (503)	53	4.37 (3 70, 5.04)	<0.00001
	>3 months	2 (115)	84	4.67 (3.51, 5.84)	<0.00001
Intervention cycle	>4 weeks	5 (218)	0	3 19 (1 75, 4.62)	<0.0001
	<4 weeks	7 (400)	68	4.69 (4 06, 5 33)	<0.00001
Single-session	≥30 min	11 (573)	56	4.28 (3.67, 4.90)	<0.00001
Intervention duration	<30 min	1 (45)		6.10 (4.15, 8.05)	
Frequency	>5 times/week	6 (335)	62	4.44 (3.65, 5.22)	<0.00001
	≤5 times/week	6 (283)	59	4.46 (3.29, 5.33)	<0.00001

### 3.7 Publication bias analysis

**Figure 7:**
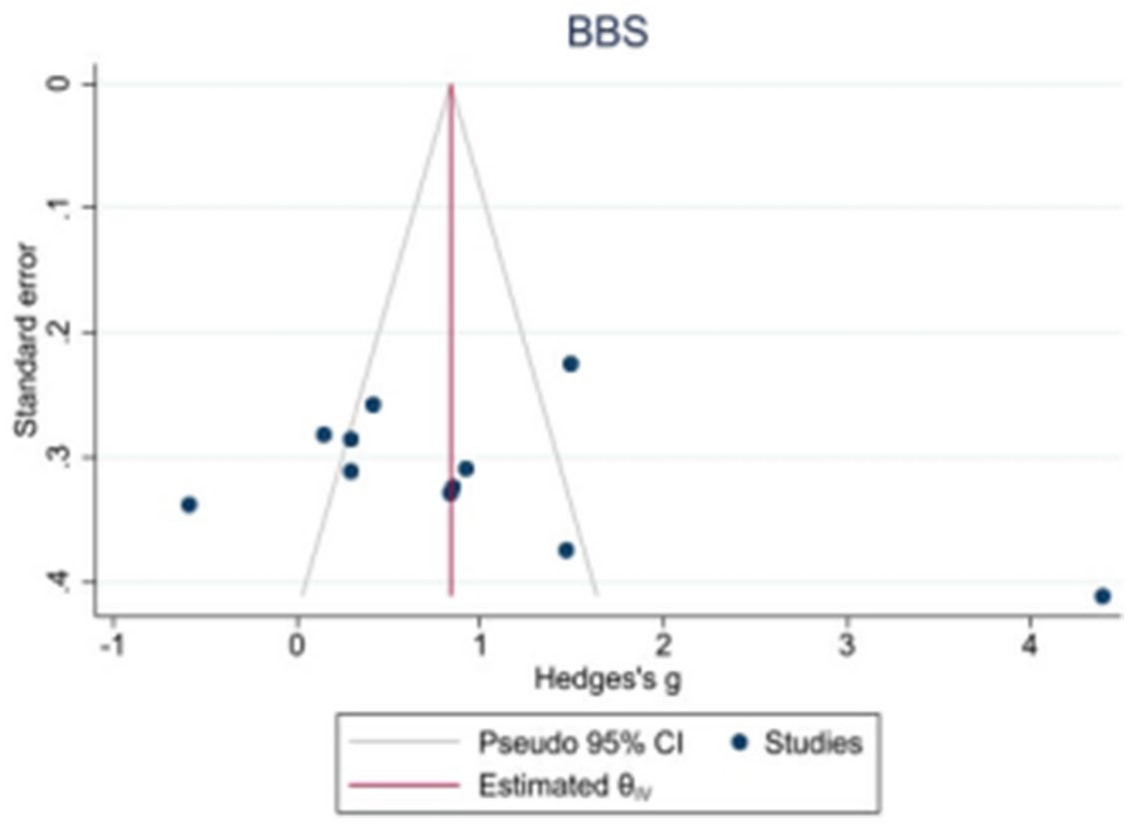
Funnel plot of potential publication bias of BBS.

**Figure 8:**
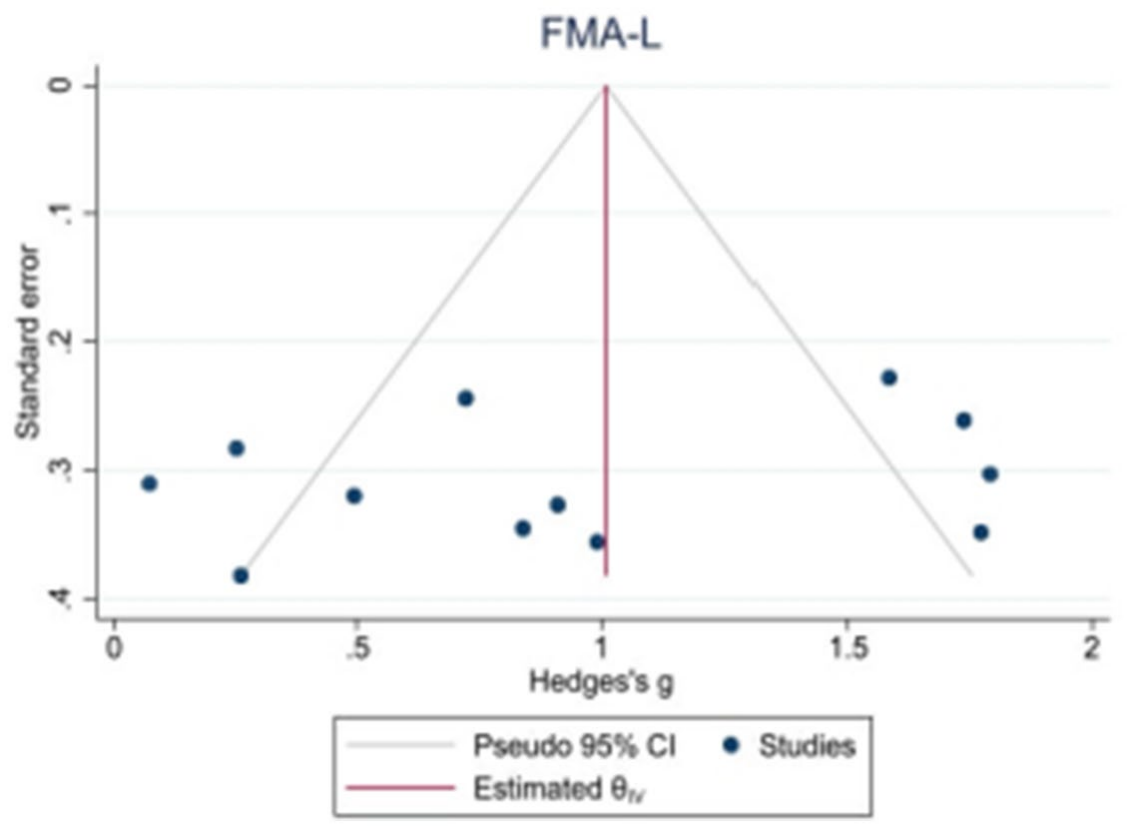
Funnel plot of potential publication bias of FMA-L.

**Figure 9:**
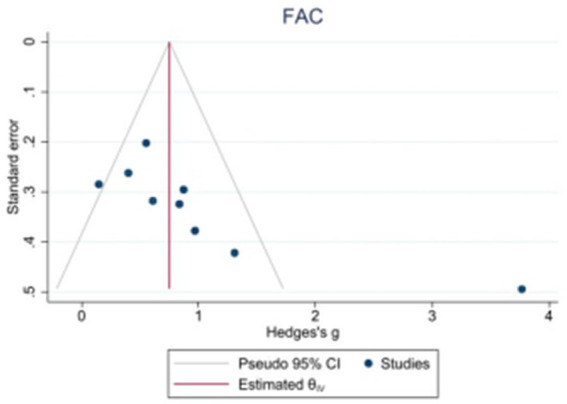
Funnel plot of potential publication bias of FAC.

Because the 10MWT and 6MWT included too few studies, only the publication bias test was performed on the BBS, FAM-L, and FAC. Egger’s test revealed that there was no obvious publication bias in BBS (|t| = 1.82, *p* > 0.05) or FAM-L (|t| = 0.89, *p* > 0.05). FAC (|t| = 3.74, *p* < 0.05) and Egger’s test results revealed publication bias (see Figures 7–9).

### 3.8 Quality assessment of evidence

The GRADEPro software indicates that the quality of evidence for lower extremity motor function is low, and for balance function, it is moderate (see Table 9).

**Table 9:** Grade evidence quality grading of major outcome indicators.

Outcome measures	Included studies	Assessment of evidence quality					Quality of evidence
		Study bias	Inconsistency	Indirectness	Imprecision	Bias of publication	
Lower limb motor function	16	Serious	Not serious	Not serious	Not serious	Serious	Low
Function of balance	11	Serious	Not serious	Not serious	Not serious	Not serious	Moderate

## 4 Discussion

The present study demonstrated that end-effector robotic training may exert a positive effect on improving lower limb function in patients with stroke. Specifically, regarding balance function, the MD of the BBS score was 5.24 (95% confidence interval [CI]: 1.10–9.38, *p* = 0.01). This magnitude of improvement exceeded the minimal clinically important difference (MCID = 4 points) of the scale, suggesting potential clinical significance (38). For lower limb motor function, the pooled MD of the Fugl-Meyer Assessment of FMA-L score reached 4.06 (95% CI: 3.08–5.03, *p* < 0.00001). The FAC score increased by 0.70 grades (95% CI: 0.60–0.79), and a significant improvement was also observed in the 10MWT speed (SMD = 0.40, 95% CI: 0.14–0.67). These findings are consistent with certain research trends in the field of neurorehabilitation (39, 40); however, the impact of inter-study heterogeneity should be noted.

From the perspective of the underlying mechanism, the potential therapeutic efficacy of end-effector robots may be associated with the following factors. First, their unique plantar pressure feedback system can dynamically adjust the patient’s center of gravity displacement in real time. This closed-loop sensory input may play a role in reconstructing the neural pathways for balance control. A study by Inoue et al. confirmed that this feedback mechanism can significantly enhance the neural adaptability of postural adjustments (7). Second, the precise and repetitive gait training provided by robots may help reconstruct motor programs. Tomida et al. indicated that such training can optimize short-distance gait parameters (41), which may partially explain the improvements in FAC and 10MWT observed in this study. Third, compared with conventional training, robot-assisted training enables higher-intensity repetitive practice, which may exert a positive effect on promoting functional reorganization of the motor cortex in the brain.

### 4.1 Effects of end-effector robotic training on balance function

This potential advantage may stem from its unique sensory feedback mechanism: the end-effector device regulates center of gravity displacement in real time via plantar pressure, thereby enhancing the neural adaptability of postural adjustments (42). Subgroup analysis revealed that in terms of training parameters, high-dose training (> 5 sessions/week for > 4 weeks) appeared to yield significantly superior outcomes compared with low-dose regimens (MD = 7.00 vs. 2.40). This finding provides partial support for the “dose-dependent effect” hypothesis—i.e., within a certain range, training efficacy may increase with the elevation of training dose. These results align with the fundamental principles of neurorehabilitation, which emphasize that a sufficient training volume is a prerequisite for inducing neuroplasticity (43). In terms of patient characteristics, patients in the chronic phase (disease duration > 3 months) may exhibit greater improvement potential (MD = 10.97 vs. MD = 3.72 in the ≤ 3 months group). This phenomenon may reflect differences in neural remodeling mechanisms across different disease stages: recovery in the acute phase mainly relies on edema resolution and local blood flow improvement, whereas functional recovery in the chronic phase is more dependent on central compensation and transhemispheric reorganization (44). Notably, in a study by Wang et al., pelvic belt reinforcement was used to enhance core stability. After excluding this study, heterogeneity decreased significantly from 96 to 85%, indicating that pelvic control ability may be a crucial factor influencing BBS scores (27). This finding provides important implications for clinical practice: integrating core stability interventions into robotic training may produce a synergistic effect. Additional studies have shown that the recovery of balance function during inpatient rehabilitation is a core indicator for predicting the achievement of unassisted walking in patients (45). Research has demonstrated that robotic devices can assist patients in completing high-intensity and repetitive gait training tasks, stimulate the vestibular and proprioceptive systems during training, induce changes in muscle mass, and further potentially affect balance and lower limb function, thereby improving neuroplasticity and functional recovery in patients (42, 46). Therefore, end-effector robotic training may serve as a viable rehabilitation option for patients with balance dysfunction, but its application should be comprehensively considered based on clinical practice.

### 4.2 Effects of end-effector robotic training on lower limb motor function

Age was found to exert a certain trend of influence: patients aged ≤65 years may achieve more significant benefits (MD = 4.64 vs. MD = 4.15 in the > 65 years group). This result is consistent with the findings of Wall et al., who reported that younger patients generally exhibit higher rates of motor function recovery after stroke (47). Possible explanations include stronger neuroplastic potential, fewer comorbidities, and better training tolerance in younger patients (48). In terms of training protocols, short-term intensive training (≤ 4 weeks) appeared to demonstrate more prominent efficacy (MD = 4.69 vs. MD = 3.19 in the > 4 weeks group), which may reflect the rapid activation of neural adaptation in the early stage of training (49, 50). Interestingly, however, differences in training frequency (> 5 sessions/week vs. ≤ 5 sessions/week) did not lead to significant differentiation in effect sizes (MD = 4.46 vs. 4.44), suggesting that frequency may not be an independent influencing factor, or that a “ceiling effect” exists—i.e., beyond a certain threshold, increasing training frequency may fail to yield additional benefits.

Studies have indicated that lower limb function in patients can be improved through gait training (51, 52), which is consistent with the results of the present study. However, it should be noted that no statistically significant difference was observed between the two groups in the 6MWT, an indicator of walking endurance (MD = 22.92, 95% CI: −38.98–84.42, *p* = 0.47). Moreover, this outcome measure was based on data from only 2 trials, indicating limited evidence strength. This result may suggest that end-effector training has certain limitations in improving endurance-based walking capacity. A deeper analysis of the underlying reasons is as follows. On the one hand, the intensity of existing training protocols may be insufficient: studies have shown that the average intensity of end-effector training only reaches 45% of peak oxygen uptake, which is insufficient to effectively improve aerobic endurance (53). On the other hand, the kinematic limitations of such devices (e.g., inhibition of coronal plane pelvic movement) may affect the natural adaptability of gait (54). Additionally, studies have found a delay of approximately 120 ms in the activation of the medial gastrocnemius (55); this deficit in neuromuscular coordination may weaken propulsion efficiency and further limit endurance performance. A meta-analysis by Tedla et al. indicated that robot-assisted training is significantly superior to conventional therapy in improving gait symmetry (16). Studies have pointed out that leg muscle strength is a key factor affecting walking speed (56). Meanwhile, Hwang et al. confirmed that end-effector robots can enhance muscle strength while protecting muscles through weight-bearing-free training with 100% guidance force (17). However, compared with exoskeleton robots, end-effector devices may be slightly inferior in improving walking endurance. A study by Hu et al. showed that patients achieved more significant improvements in 6MWT after exoskeleton robotic training (39), which may be attributed to the more comprehensive limb support provided by exoskeletons. It should be noted that potential uncertainty may exist in the classification of robot types in the present study, which may also affect the analysis results of relevant outcomes. Therefore, although end-effector robots may help improve walking speed and functional ambulation ability in patients, their efficacy in enhancing endurance is suboptimal. In clinical practice, rehabilitation programs should be selected comprehensively based on the specific needs of patients, device characteristics, and potential classification limitations.

### 4.3 Sensitivity analysis and robustness evaluation of results

To verify the reliability of the study results, systematic sensitivity analyses were performed. For BBS scores, after excluding the study by Wang et al., the pooled effect size remained significant (MD = 4.01, 95% CI: 1.46–6.57), and heterogeneity decreased significantly, confirming that pelvic control interventions may introduce special confounding factors. FMA-L scores exhibited good stability: after excluding any individual study, the effect size ranged from 3.82 to 4.32, indicating low sensitivity. The I^2^ of 10MWT fluctuated between 25 and 54% after excluding each study, with no significant changes, suggesting that this result has good reproducibility. However, it should be noted that high heterogeneity persists in the overall study, which may affect the robustness of the results.

### 4.4 Limitations and future directions

First, there is substantial methodological heterogeneity: differences exist in training protocols (e.g., single-session duration, total course of treatment) and robot models used in included studies, which may affect the reliability of the results. Second, direct neurophysiological evidence (e.g., electromyography, functional magnetic resonance imaging data) is lacking to clarify the intrinsic mechanisms underlying functional improvements. Third, due to the limitations of original data, more refined subgroup analyses (e.g., by stroke severity or robot type) could not be performed. In addition, some outcome measures (e.g., 6MWT) were based on a limited number of trials (only 2), resulting in insufficient evidence strength. Fourth, none of the included studies tracked long-term efficacy (> 12 weeks), making it impossible to evaluate the sustainability of treatment effects. Fifth, there may be a potential risk of misclassification of robot types, which may affect the accuracy of the analysis results.

To address the existing limitations, future research should focus on the following directions: (1) Conduct multicenter, large-sample standardized randomized controlled trials (RCTs) with strict control of training parameters to reduce heterogeneity; (2) Combine neuroimaging and electrophysiological techniques to explore therapeutic mechanisms in depth; (3) Develop precision training protocols tailored to different clinical subgroups (e.g., different severities and disease stages); (4) Perform long-term follow-up to assess the maintenance of functional improvements; (5) Explore the synergistic effects of robotic training combined with other interventions (e.g., transcranial magnetic stimulation, biofeedback); (6) Optimize the classification criteria for robot types to reduce the impact of classification uncertainty on research results.

## 5 Conclusion

This GRADE systematic review (moderate-quality evidence for balance, low-quality for lower limb motor function) preliminarily concludes that end-effector robot-assisted training may improve stroke patients’ balance (with definitive clinical significance) and lower limb motor function. Training efficacy is modulated by dosage and patient characteristics; however, it has limited effect on walking endurance, requiring combination with other therapies. Personalized programs are recommended given the intervention’s strengths and evidence limitations.

## Data Availability

The original contributions presented in the study are included in the article/supplementary material, further inquiries can be directed to the corresponding author.
